# Patients’ Preferences for Information About the Benefits and Risks of Second-Line Palliative Chemotherapy and Their Oncologist’s Awareness of These Preferences

**DOI:** 10.1007/s13187-015-0845-9

**Published:** 2015-05-20

**Authors:** Linda J. M. Oostendorp, Petronella B. Ottevanger, Agnes J. van de Wouw, Aafke H. Honkoop, Maartje Los, Winette T. A. van der Graaf, Peep F. M. Stalmeier

**Affiliations:** 1 Department for Health Evidence, Radboudumc, PO Box 9101, 6500 HB Nijmegen, The Netherlands; 2 Department of Medical Oncology, Radboudumc, Nijmegen, The Netherlands; 3 Department of Internal Medicine, VieCuri Medical Centre, Venlo, The Netherlands; 4 Department of Internal Medicine, Isala Clinics, Zwolle, The Netherlands; 5 Department of Internal Medicine, St. Antonius Hospital, Nieuwegein, The Netherlands

**Keywords:** Breast cancer, Colorectal cancer, Palliative chemotherapy, Information preference, Decision aids

## Abstract

Communication about palliative treatment options requires a balance between providing patients with sufficient information and not providing unwanted information. Surveys have indicated that many patients with advanced cancer express a wish to receive detailed information. In this prospective multicenter study, the information desire of patients with advanced breast or colorectal cancer was further investigated by offering treatment-related information to patients using a decision aid (DA). In addition, this study explored oncologists’ awareness of their patients’ information desire. Seventy-seven patients with advanced breast or colorectal cancer facing the decision whether to start second-line palliative chemotherapy were offered a DA by a nurse. This DA contained information on adverse events, tumor response, and survival. The nurse asked the patient whether each information item was desired. Ninety-five percent of patients chose to receive information on adverse events, 91 % chose to receive information on tumor response, and 74 % chose to receive information on survival. Oncologists’ judgment of patients’ information desire was 100, 97, and 81 %, respectively. For all three information items together, oncologists correctly judged the information desire of 62 % of patients. This study confirms that many patients with advanced cancer wish to receive detailed information on the benefits and risks of palliative treatment options when the information is actually available. Oncologists were adequately aware of this high information desire, but had some difficulty judging the information desire of individual patients. A stepped approach to giving information (“preview, ask, tell, ask”) may help to better meet patients’ information needs.

**Electronic supplementary material:**

The online version of this article (doi:10.1007/s13187-015-0845-9) contains supplementary material, which is available to authorized users.

## Introduction

Palliative chemotherapy aims at prevention and relief of disease symptoms. The impact of treatment on length of life is often modest or uncertain, and the occurrence of adverse events can negatively impact quality of life. Therefore, the decision whether to start palliative chemotherapy involves a personal trade-off between the potential benefits and risks of treatment [1]. When informing patients about this treatment option, information provision needs to be balanced in order to meet informed consent, while not providing any unwanted information [2].

In daily clinical practice, achieving this balance may be difficult. Findings from surveys suggest that many patients in the palliative treatment setting wish to receive detailed treatment-related information [3, 4]. Little is known about the ability of physicians to judge patients’ information desire. One study in the palliative setting showed that physicians had difficulty predicting patients’ stated preferences for information on expected survival [5]. Likewise, studies on treatment decision-making showed poor concordance between patients’ preferences and physicians’ perceptions of these preferences [5–9]. Reference has been made to the “silent misdiagnosis” of patients’ treatment preferences [10].

The present study focused on the treatment decision whether or not to start second-line palliative chemotherapy. Beyond first-line treatment for advanced disease, benefits of subsequent lines of chemotherapy are reduced and also often less clear, emphasizing the preference-sensitive nature of this decision. Furthermore, the study focused on two common types of cancer, i.e., colorectal and breast cancer. Unlike previous studies using hypothetical scenarios, patients’ information desire was assessed by actually offering information about the benefits and risks of treatment options to patients, using a decision aid (DA). The aim of the study was to investigate patients’ desire for information about the benefits and risks of second-line treatment, and explore their oncologists’ awareness of this desire.

## Methods

### Study Design

The study described here was part of a multicenter randomized trial; this trial was registered in the Netherlands Trial Registry (NTR1113), and details have been published in the study protocol [11]. In short, the target population consisted of patients with advanced breast or colorectal cancer facing the treatment decision whether or not to start second-line palliative chemotherapy. To identify these patients, we recruited patients who had started or were starting first-line palliative chemotherapy for advanced breast or colorectal cancer. Exclusion criteria were labile personality structure (as assessed by the physician), a Karnofsky performance score lower than 60, and insufficient knowledge of the Dutch language. The study was approved by the regional ethics review committee (CMO Arnhem-Nijmegen) and the research ethics committees of all participating centers.

The medical oncologist or nurse assessed the potential eligibility of consecutive patients. Health professionals were instructed to introduce the study topic as to “how to involve the opinion of patients in their treatment.” Patients were not to be told that detailed risk information (e.g., on survival) could be provided in this study, to avoid selection of patients based on information desire. Health professionals asked patients for permission to be approached by the researcher, who obtained written informed consent.

After inclusion, patients were monitored for disease progression and the subsequent decision whether or not to start second-line palliative chemotherapy. Patients who were proposed second-line treatment were randomly assigned to receive (1) the usual treatment-related information from the oncologist (control group) or (2) the usual treatment-related information from the oncologist plus a DA with information from a nurse (intervention group) in a 1:2 ratio. Treatment allocation was concealed using sealed envelopes which were opened by the nurse after the oncologist mentioned disease progression and offered second-line treatment. Unequal randomization was employed because the control group was only needed to evaluate the DAs (results of the randomized comparison are reported in a separate manuscript), while data from the intervention group were also used to address questions on patients’ information desire [11].

### Outcome Measures

For each patient included in the study, the oncologist completed an inclusion form with patient and disease characteristics, and stated a judgment of whether the patient would desire information for each of the three items in the DA: (1) adverse events, (2) tumor response, and (3) survival. Patients completed a baseline questionnaire on sociodemographic variables.

When disease progression occurred and the oncologist had proposed second-line chemotherapy, patients in the intervention group received the DA in a subsequent consultation with a nurse, typically within a week. The data on risks and benefits presented in the DA were obtained from systematic reviews of the literature for the two tumor types [12, 13]. DAs were developed for 11 chemotherapeutic regimens commonly used as second-line treatment for patients with advanced breast or colorectal cancer. All DAs were reviewed and approved by the participating oncologists. An example of a DA for colorectal cancer is available in an online supplement.

Patients’ information desire was obtained as follows. Using the DA, information on the three items was presented in a stepwise fashion (see the online supplement). The nurse first explained the type of information that could be expected and then asked the patient whether the information item was desired or not. If desired, the information was provided. The nurse asked the patient for each item whether any information on that item had been provided by the oncologist, to explore whether patients’ information desire was associated with the perception of previously having received information on these items from the oncologist.

### Statistical Analysis

Descriptive statistics were used to summarize patients’ information desire and oncologists’ judgment. Concordance between these two outcome measures for each of the three information items was examined by calculating the percentage of overall concordance, positive agreement (concordance for wanting the information), and negative agreement (concordance for not wanting the information), as has been suggested by Cicchetti and Feinstein [14]. To obtain more insights in the number of patients whose information desire for all three of the items was correctly judged by their oncologist, we calculated concordance for all three information items on the level of the individual patient. Associations between patients’ information desire and perceptions of having previously received information from the oncologist were explored using chi-square tests. All analyses were performed using SPSS version 20.

## Results

### Participating Patients and Oncologists

**Fig. 1:**
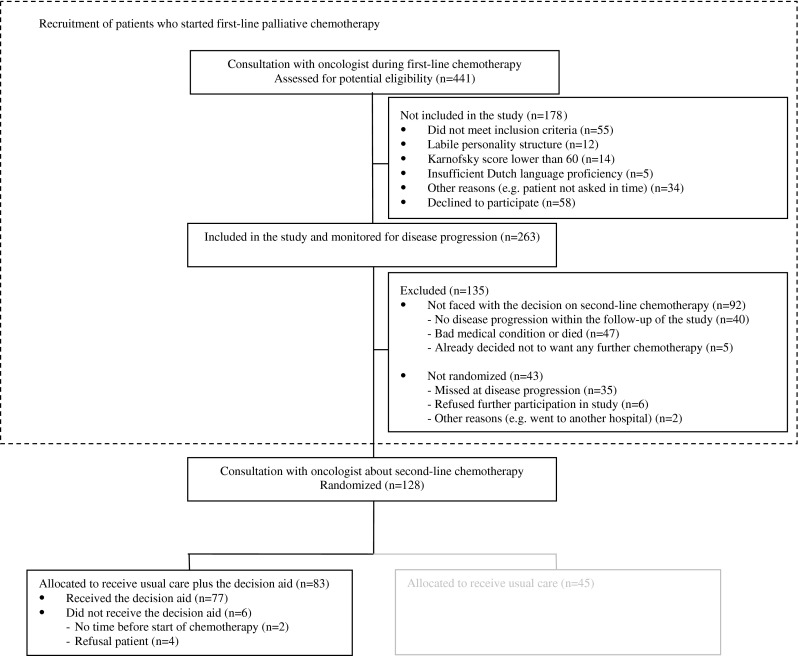
Patient flow chart

Patient flow is depicted in Fig. 1. Out of 441 patients assessed for potential eligibility, 55 (12 %) did not meet the inclusion criteria, 31 (7 %) were excluded, and 34 (8 %) were not approached by the oncologist, and therefore, the inclusion criteria could not be verified. Of the 321 patients approached to participate in this study, 263 (82 %) gave informed consent. From this group, 92 patients (35 %) were not faced with the decision on second-line chemotherapy and therefore did not belong to the target population of this study. Another 43 patients (16 %) faced the treatment decision but were not randomized and dropped out of the study. Of the 128 patients who were randomized, 83 patients were randomized to the intervention group, and 77 (93 %) completed the intervention interview with the nurse using the DA. Oncologists’ judgment of information desire was available for 74 of these patients. Table 1 lists the characteristics of these 74 patients and the 40 participating oncologists from 17 hospitals.

**Table 1:** Patient and oncologist characteristics

Patient characteristics (*n* = 74)	
Male sex, *n* (%)	29 (39 %)
Age, median (range)	62 (32–80)
Living with partner, *n* (%)	57 (77 %)
Employed^a^, *n* (%)	20 (40 %)
College education or more, *n* (%)	21 (28 %)
Colorectal cancer (vs. breast cancer), *n* (%)	57 (77 %)
Information preference, mean (SD)^b^	8.6 (1.6)
Oncologist characteristics (*n* = 40)	
Male sex, *n* (%)	18 (45 %)
Academic hospital (vs. peripheral), *n* (%)	13 (33 %)
Number of judgments made per oncologist, mean (range)	2 (1–8)

*SD* standard deviation

^a^Selection of patients <65 years of age, retirement age in the Netherlands in 2012

^b^Rating scale from 0 (I want to know nothing about the illness and its treatment) to 10 (I want to know everything there is to know about the illness and its treatment)

### Information on Adverse Events, Tumor Response, and Survival

During the interview, 95 % of patients chose to receive information on adverse events, 91 % chose to receive information on tumor response, and 74 % chose to receive information on survival. There were no differences in information desire between patients who perceived or did not perceive to have received any information on this topic from the oncologist. Oncologists thought that of 74 patients, all would want information on adverse events (100 %), 97 % would want information on tumor response, and 81 % would want information on survival.

### Concordance Between Oncologists’ Judgment and Patients’ Information Desire

Table 2 shows the concordance between oncologists’ judgment and patients’ information desire. Depending on the item, oncologists correctly judged the information desire of 47 to 70 out of 74 patients (64–95 %). Positive agreement for wanting to see information on adverse events and tumor response was 97 and 94 %, respectively, and 77 % for wanting to see information on survival. Negative agreement for not wanting to see information on the three items ranged between 0 and 18 %. On the level of the individual patient, oncologists’ judgments were concordant with the information desire of 46 patients (62 %) for all three information items.

**Table 2:** Oncologists’ judgment versus patients’ actual information desire

	Patients’ desire (*n* = 74)		Overall concordance (%)	Positive agreement^a^ (%)	Negative agreement^b^ (%)
	Wanted information	Did not want information			
Item 1: adverse events					
Oncologists’ judgment (*n* = 74)					
Wants information	70^c^	4			
Does not want information	0	0^c^			
Concordance			95	97	0
Item 2: tumor response					
Oncologists’ judgment (*n* = 74)					
Wants information	65^c^	7			
Does not want information	2	0^c^			
Concordance			88	94	0
Item 3: survival					
Oncologists’ judgment (*n* = 74)					
wants information	44^c^	16			
does not want information	11	3^c^			
*concordance*			64	77	18

^a^Positive agreement (concordance for wanting the information) = 2*a*/(2*a* + *b* + *c*), where *a* represents the cases where oncologists correctly judged that patients wanted to receive the information, *b* represents the cases where oncologists judged that patients would want the information but the patients did not want to receive it, and *c* represents the cases where patients wanted the information but oncologists judged they would not want to

^b^Negative agreement (concordance for not wanting the information) = 2*d*/(2*d* + *b* + *c*), where *b* represents the cases where oncologists judged that patients would want the information but the patients did not want to receive it, *c* represents the cases where patients wanted the information but oncologists judged they would not want to, and *d* represents the cases where the oncologists correctly judged that the patient would not want to receive the information

^c^Overall concordance

## Discussion

This study sought to determine whether the high information desire as stated by patients with advanced cancer in surveys would also hold true when actually offering treatment-related information in a DA. The findings confirm that almost all patients with advanced breast or colorectal cancer wished to receive information on adverse events and tumor response rates, and that three quarters of patients wished to receive detailed survival information related to second-line treatment options.

The oncologists in this study were adequately aware of their patients’ high information desire. They were generally capable of identifying patients wanting information about adverse events and tumor response, but had more difficulty identifying patients wanting information about survival and patients not wanting the available information. These results are in line with the previously described “silent misdiagnosis” of patients’ information preferences [5], and treatment and participation preferences [5–10].

The majority of patients in this study wished to receive all available information from the DA, including detailed survival information. However, audio-recordings of consultations on first-line palliative chemotherapy have shown that 61 % of patients are not given survival information [15]. In addition, a systematic review illustrated that communication on prognosis is characterized by lack of clarity, lack of an estimate of expected survival, and avoidance of this topic by focusing on active treatment options [16]. Perhaps not surprisingly, studies have shown that more than two thirds of patients who had started first-line palliative chemotherapy did not seem to understand that the goal of treatment was not cure [17], and that only 49 % of patients with advanced cancer were fully aware of their prognosis [4]. Oncologists might be hesitant to impart survival information, possibly fearing a negative impact on the patient [18]. Such fear, however, may not be warranted. For instance, in patients with advanced cancer, full prognostic discussion was associated with lower levels of depression and did not impact on anxiety [19]. In the same vein, prognostic information could be provided to patients without taking away hope [20], and end-of-life discussions did not inflict psychological distress [21]. On the contrary, not discussing prognosis may cause distress and may preclude patients from reorganizing and adapting their lives [22]. It was also found that patients reporting not having had end-of-life discussions received more aggressive medical care near death and later hospice referral, resulting in worse quality of life [21].

What can be done to help clinicians to better meet patients’ desire for information? Our suggested approach for clinicians is to start discussions by eliciting the patient’s information desire by using open-ended questions [23]. In addition, a stepped approach to giving information, as employed in this study, could help to clarify a patient’s information desire. This approach includes three steps: (1) giving the patient a preview of the type of information available, (2) asking whether the information is desired, and (3) then following the patient’s desire. This stepwise approach is consistent with recommendations on the communication of prognosis to patients with advanced disease [23]. DAs can help to implement this approach by providing numerical estimates of benefits and risks of treatment options; previous studies showed that DAs can improve understanding [24]. The clinician can further promote understanding by asking what the patient understands and feels about the information provided [25].

Methodological strengths of our study are that information desire was assessed by providing treatment-related information to patients at the point of decision-making. Generalizability is facilitated by the recruitment of patients using broad inclusion criteria, before the moment of disease progression. To prevent selective patient participation, physicians were instructed to recruit consecutive patients and not to mention that detailed risk information could be provided. This study has a number of limitations. Ideally, oncologists’ judgment of their patients’ information desire would be recorded between the diagnosis of disease progression and the treatment discussion with the patient. However, recording oncologists’ judgment in this narrow window of opportunity was expected to result in a large amount of missing data. Asking oncologists for a retrospective judgment, as was done in a previous study on palliative treatment decisions [5], would introduce the risk of recall bias. We therefore decided to record the oncologists’ judgment beforehand (median time of 5 months before the treatment discussion), knowing that most oncologists in this study had a long-standing relationship with their patients (as an indication, median time since first diagnosis of disease and diagnosis of metastatic disease were 23 and 10 months, respectively). While our findings are in line with those of previous studies, they will need to be confirmed by further studies of real-world treatment decisions. It is also worth noting that the accuracy of predicting patient’s desire for survival information may vary between doctors, but this could not be analyzed due to the small number of judgments made per doctor. In hindsight, it would have been worthwhile to also ask the patients in the control arm of the randomized trial about their information desire, without offering the information, to examine the extent to which patients’ information desire is dependent on the context in which it is assessed.

In conclusion, this study confirms that many patients with advanced cancer wish to receive detailed information on the benefits and risks of palliative treatment options. Oncologists were aware of this high information desire, but had some difficulty judging the information desire of individual patients. A stepped approach, possibly facilitated by the use of DAs, may help to better meet patients’ information needs.

## Electronic Supplementary Material

Below is the link to the electronic supplementary material.

ESM 1 (DOCX 13 kb)
