# Supplementary material for: Patients’ Preferences for Information About the Benefits and Risks of Second-Line Palliative Chemotherapy and Their Oncologist’s Awareness of These Preferences
Source: J Cancer Educ. 2015 May 20;31:443–8. doi: 10.1007/s13187-015-0845-9 (PMC4988994; doi:10.1007/s13187-015-0845-9)
Supplement: Supplementary file 1 — (DOCX 13 kb) [file 13187_2015_845_MOESM1_ESM.docx]

| Introduction |
| --- |
| The nurse introduces the decision aid |
| The nurse introduces the aim of treatment and the two treatment options:best supportive carebest supportive care plus second-line chemotherapy |
| The nurse gives a non-health related example of the presentation of risk information |
| Information |
| Adverse events |
| The nurse explains the implications of a severe adverse event (e.g. hospital admission), and explains the selection of adverse events that has been made |
| The nurse asks the patient whether the information on severe adverse events is desired |
| If desired, the nurse provides the information |
| Tumour response |
| The nurse explains the different ways in which the tumor can respond to treatment, including the temporary nature of this response |
| The nurse asks the patient whether the information on tumor response is desired |
| If desired, the nurse provides the information |
| Survival |
| The nurse explains the concept of median survival, and emphasizes that it is not possible to predict an individual patient’s survivalThe nurse asks the patient whether the information on expected survival is desiredIf desired, the nurse provides the information |
| Wrap-up |
| The nurse explains that the information can be used to make a treatment choice |
| The nurse gives the patient a brochure with the information the patient desired |

**Online supplement.** Interview with the nurse using the decision aid
